# Identifying natural inhibitors against FUS protein in dementia through machine learning, molecular docking, and dynamics simulation

**DOI:** 10.3389/fninf.2024.1439090

**Published:** 2025-02-05

**Authors:** Darwin Li

**Affiliations:** Computer Science and Design Department, Upper Canada College, Toronto, ON, Canada

**Keywords:** dementia, FUS protein, machine learning, molecular docking, molecular dynamic simulation

## Abstract

Dementia, a complex and debilitating spectrum of neurodegenerative diseases, presents a profound challenge in the quest for effective treatments. The FUS protein is well at the center of this problem, as it is frequently dysregulated in the various disorders. We chose a route of computational work that involves targeting natural inhibitors of the FUS protein, offering a novel treatment strategy. We first reviewed the FUS protein's framework; early forecasting models using the AlphaFold2 and SwissModel algorithms indicated a loop-rich protein—a structure component correlating with flexibility. However, these models showed limitations, as reflected by inadequate ERRAT and Verify3D scores. Seeking enhanced accuracy, we turned to the I-TASSER suite, which delivered a refined structural model affirmed by robust validation metrics. With a reliable model in hand, our study utilized machine learning techniques, particularly the Random Forest algorithm, to navigate through a vast dataset of phytochemicals. This led to the identification of nimbinin, dehydroxymethylflazine, and several other compounds as potential FUS inhibitors. Notably, dehydroxymethylflazine and cleroindicin C identified during molecular docking analyses—facilitated by AutoDock Vina—for their high binding affinities and stability in interaction with the FUS protein, as corroborated by extensive molecular dynamics simulations. Originating from medicinal plants, these compounds are not only structurally compatible with the target protein but also adhere to pharmacokinetic profiles suitable for drug development, including optimal molecular weight and LogP values conducive to blood-brain barrier penetration. This computational exploration paves the way for subsequent experimental validation and highlights the potential of these natural compounds as innovative agents in the treatment of dementia.

## 1 Introduction

Dementia represents the most prominent reason behind the defects associated with the individual's cognitive functions that are mostly related to aging making them completely dependent to caretakers. Almost 55 million people are found to be suffering from dementia, and this number is expected to be doubled by the year 2050 (GBD 2019 Dementia Forecasting Collaborators, 2022). Patients suffering from dementia face difficulties in controlling their emotions and social behavior and interaction with other people (Emmady and Tadi, 2022). Certain neurodegenerative disorders that are the closely linked to dementia include Alzheimer's disease, lewy body dementia and frontotemporal dementia, where the most significant cases arising due to Alzheimer disease i.e., 75% and the other representing 5% to 10% of all dementia patients (Qiu et al., 2009). Some other diseases contributing to dementia include Parkinson disease, vascular dementia due to brain injury, alcohol, and smoking affecting brain. The complex mechanism behind the development of dementia remains largely unknown and the only assumption behind the development of dementia include deposition of certain proteins in affecting the normal functioning of brain thus causing behavioral changes and speaking abnormality (Zagórska et al., 2023).

Accumulation of α-synuclein in neuronal cells lead to the development of lewy body dementia and the development of dementia related psychosis and impairment in visuospatial function (Garcia-Esparcia et al., 2017). Frontotemporal dementia characterization has been made by the accumulation of subsequent disordered proteins in gray and white matter of brain (De Conti et al., 2017). Cerebral ischemia and hemorrhagic injury to the certain tissue can lead to vascular dementia (Kalaria, 2016).

The diagnosis for dementia is based on the patient history of impairment in daily activities and cognitive decline following the investigation by close friend or family members along with in detail examination by physician to get further confirmation. Brain neuroimaging can also be referred for detection of changes in brain structure (Arvanitakis et al., 2019).

There are certain FDA approved drugs to minimize the effects of dementia related disorders in brain. Donepezil, galantamine, and rivastigmine are the drugs that are currently being utilized in treatment of dementia (Marucci et al., 2021). There is still no FDA approved drug has been available for lewy body dementia. However, the purpose of each medication is to treat the dementia related symptoms and no drug particularly targets dementia (Zagórska et al., 2023).

The target-based drug designing approach has been utilized by most of the researchers to find the most probable drug targets against a particular disease but with confounding increase in multi-omics data there is need to bridge the gap between drug discovery and development against a particular disease via machine learning (Doherty et al., 2023). To explore the dementia linkage to the genetic and abnormal changes in brain the most viable method is to utilize machine learning based approaches for identification of therapeutic targets against dementia.

The present study focuses on the identification of natural inhibitors against FUS protein in dementia by utilizing machine learning approaches then validating the potential targets by molecular docking and dynamic simulation studies. Further studies on the development of inhibitors against the FUS protein would be increasingly helpful in near future to develop effective drugs.

## 2 Methods

### 2.1 Structure prediction of FUS protein

The FUS proteins three dimensional (3D) structure was not present, in the RCSB Protein Data Bank (PDB). Hence three different programs I-TASSER (https://zhanggroup.org/I-TASSER/) (Zhang, 2008), SwissModel (https://swissmodel.expasy.org/) (Schwede et al., 2003), and AlphaFold2 (https://colab.research.google.com/github/sokrypton/ColabFold/blob/v1.1-premultimer/AlphaFold2.ipynb) (Cramer, 2021) were used to predict the proteins structure. These tools were chosen for their proven effectiveness in predicting protein structures. Following the predictions a thorough quality assessment was carried out to gauge the reliability and accuracy of the models generated. The assessment involved steps. Initially the overall structural quality was evaluated using the ERRAT score, which measures alignment with expected parameters quantitatively. Subsequently hydrogen atoms were incorporated into the 3D structures using UCSF Chimera (Huang et al., 2014) to improve accuracy for analyses. Additionally, the proteins structural integrity was examined through a Ramachandran plot to gain insights, into backbone torsion angle distribution.

### 2.2 Machine learning-based virtual screening of natural compounds against FUS protein

#### 2.2.1 Data collection

After conducting an extensive literature review to gain insights into the molecular interactions associated with FUS protein, the resulting dataset described in binary format was obtained. Overall, it included a set of 463 active molecules with distinct activity toward FUS proteins considered as “1” and a set of 575 decoys containing either inactive or undetermined for FUS protein interaction molecules considered as “0.” Thus, the compiled dataset includes a variety of 1,038 entity compendium. The dataset consolidation process was carried out systematically and was designed to support machine learning ML frameworks and further use for prospective virtual drug screening. Thus, the dement dataset comprises a wide scope of molecules suitable for the development, configuration, and efficacy-testing of predictive models associated with possible interactions of FUS protein.

#### 2.2.2 Molecular descriptor generation

Following the collection of the FUS-targeted chemical compounds and proteins in Section 1.1, the dataset was processed further in a Python computational environment where the molecules were defined as pandas DataFrames structured around a Simplified Molecular Input Line Entry System using a SMILES notation. The SMILES were label “activity” as a descriptor for each chemical entity depending on its effect on FUS protein. In the course of different analysis, this data needed to be split up into predictors and responses. For effective learning and evaluation on algorithms which will follow the data was split into training 80% of the all available entities and test 20% datasets using Scikit-learn package's train_test_split function. The balance on the amounts of active and inactive entities had to be maintained. The SMILES notations were converted into characteristics. Using the RDKit library (Lovrić et al., 2019) each entity was assigned a set of 33 molecular attributes, including properties, like LogP, Molecular Weight (MW) and several others.

### 2.3 Implementation and evaluation of ML models

In the study a thorough classification process was used on the data to distinguish between inactive compounds. Various machine learning algorithms, like Support Vector Machine (SVM) (Pisner and Schnyer, 2020), K-Nearest Neighbors (KNN) (Zhang, 2016), Naive Bayes (NB) (Webb et al., 2010), and Random Forest (RF) (Rigatti, 2017) were applied. The use of cross validation and grid search methods, with Scikit learn played a role in improving the models effectiveness. SVM's various applications involve regression, as well as linear and non-linear classifications. We implemented the svm.SVC() function. We set the “gamma” parameter value to ‘scale' to align the sensitivity of the model with the dataset's features. A grid search was initiated for both “linear” and “rbf” kernels. KNN works on the principle of distances in the feature space. We applied the neighbors.KNeighborsClassifier() function. We tested different neighborhood sizes using a range of one to ten.

The Naive Bayes model is based on Bayes' theorem and assumes that the features are independent of each other. We incorporated the naive_bayes.GaussianNB() function. NB does not require hyperparameter tuning. Random Forest is an ensemble methodology, which integrates many decision trees. We imported the ensemble.RandomForestClassifier(). Initially, we set the number of trees in the random forest to 100. A grid search was conducted to explore the potential range of forest sizes, from 50 to 200, setting “random_state” to 1 for reproducibility. Validation: After model implementation, model training was validated using a 5-fold cross-validation to guarantee the model's performance stability irrespective of the data splits. For hyperparameter optimization, GridSearchCV was used. Different evaluation metrics such as recall, precision, F1-score, accuracy, and AUC-ROC were used to analyze the model performance, whereas AUC-ROC places more emphasis on the model's discriminative power in distinguishing active compounds from inactive ones. The final model was serialized using Python's pickle module. This process converts the trained model to a file format that is storable and can be used for future predictions, avoiding the unnecessary retraining of the model.

### 2.4 Re-screening with FDA approve-drugs

On the basis of the serialized model, we aimed to validate the model on a novel set of 12,903 phytocompounds, retrieved from databases such as PubChem (Wang et al., 2009), ChEMBL (Gaulton et al., 2012), and ZINC (Irwin and Shoichet, 2005). These compounds underwent similar preprocessing as in our original training set. The compounds were classified using the serialized model. We further refined putative therapeutics by checking for violation of Lipinski's Rule of Five and focusing on compounds with QED scores over 0.8 as potential FUS interactors.

### 2.5 Molecular docking analysis

In order to assess the affinity of drugs for target proteins, molecular docking analysis was carried out. This approach verifies the involvement of major target protein with drugs and thus helps in identifying possible drug combinations that can have synergetic effects in disease therapy. This study used AutoDock Vina 1.1.2 tool in PyRx (Dallakyan and Olson, 2015) 0.8 to dock hub proteins' predicted x-ray crystal structures against drugs' active ingredients as described by Dallakyan and Olson (2015). The compound were obtained from Pubchem database in SDF formats then processed through OpenBabel incorporated within PyRx for energy minimization purposes; while maintaining a stable conformation using Universal Force Field (UFF) and conjugate gradient descent optimization algorithm. The energy minimization procedure ran up to 2,000 steps after which it stopped when the difference in energy was <0.01 kcal/mol After minimizing the energy, the ligands were converted into pdbqt format for docking. Online CASTp tool was applied to identify binding pockets of target proteins. PyRx 0.8 was employed in performing afterwards the target docking approach to determine the binding energies between ligand molecules and target proteins. For evaluation of protein-compound interactions, Autodock Vina provides an empirical scoring function that sums up contributions from different factors. The complex with the lowest RMSD was taken as the most optimal one in terms of their ability to bind the protein. Energy values were then calculated based on their affinities with <-5.00 kcal/mol indicating strong binding and values below −7.00 kcal/mol signifying a very high affinity. Discovery Studio, PyMOL, and ChimeraX are some of the software tools used for this purpose.

### 2.6 Molecular dynamic simulations

Molecular Dynamic (MD) simulations are a complete atomic representation of molecular dynamics and are essential for understanding intricate biological interactions. GROMACS 2018 software (Van Der Spoel et al., 2005) were applied in this study to simulate the dynamics of docked complexes. Specifically, the GROMACS simulations were conducted with a time step of 2 fs, over a total duration of 100 ns. The temperature was maintained at 300 K using the Berendsen thermostat, and pressure control was achieved through the Parrinello-Rahman barostat. We selected the OPLS-AA/L force field due to its reliability in accurately modeling protein-ligand interactions, especially for systems like ours involving small molecules interacting with proteins. The simulations started with initial structures obtained from the protein's 3D structure, which were optimized using DockPrep. In beginning MD simulations, we used the complex that exhibited highest binding affinity with their initial positions as per docked states. SwissParam web server was used to parameterize ligand molecules. These simulations followed protocols based on previous studies over a 20 ns span. Various parameters such as radius of gyration, RMSD (Root Mean Square Deviation), Rog (Radius of Gyration), and Root Mean Square Fluctuation (RMSF) were employed to evaluate stability and interaction dynamics of each complex.

## 3 Results

### 3.1 3D structure of FUS protein

The FUS protein structure was initially predicted using AlphaFold2 and SwissModel, two well-established tools in the field of protein structure prediction. AlphaFold2 is renowned for its state-of-the-art performance in predicting protein structures with remarkable accuracy, as demonstrated in the Critical Assessment of protein Structure Prediction (CASP) competition (Cramer, 2021). It excels in predicting structures based on deep learning algorithms, particularly when homologous sequences are available. SwissModel, on the other hand, is a homology-based tool that generates models by aligning the target protein with known structures in the Protein Data Bank (PDB). It is highly reliable when template structures are available, offering an easy-to-use interface for structure prediction (Schwede et al., 2003).

However, in the case of the FUS protein, both AlphaFold2 and SwissModel produced models with significant loop regions. While loop regions are common in proteins, they often indicate areas of flexibility or structural uncertainty that can compromise the overall model accuracy, especially when homologous templates or sufficient evolutionary data are lacking. The ERRAT and Verify3D scores for these initial models were below acceptable thresholds, raising concerns about the reliability of these predictions. Specifically, the low Verify3D scores suggested that many residues had improper 3D environments, and the ERRAT results indicated deviations from the expected chemical environment for well-folded proteins.

To overcome these limitations, we turned to I-TASSER (Iterative Threading ASSEmbly Refinement), which uses a more comprehensive approach to protein structure prediction. Unlike AlphaFold2 and SwissModel, which rely heavily on deep learning or homology-based modeling, I-TASSER integrates multiple threading alignments with iterative structural assembly simulations. This allows I-TASSER to refine predicted structures by assembling fragments from similar proteins, even when homologous templates are absent or incomplete (Zhang, 2008). For the FUS protein, I-TASSER generated a model with significantly improved structural features.

**Figure 1:**
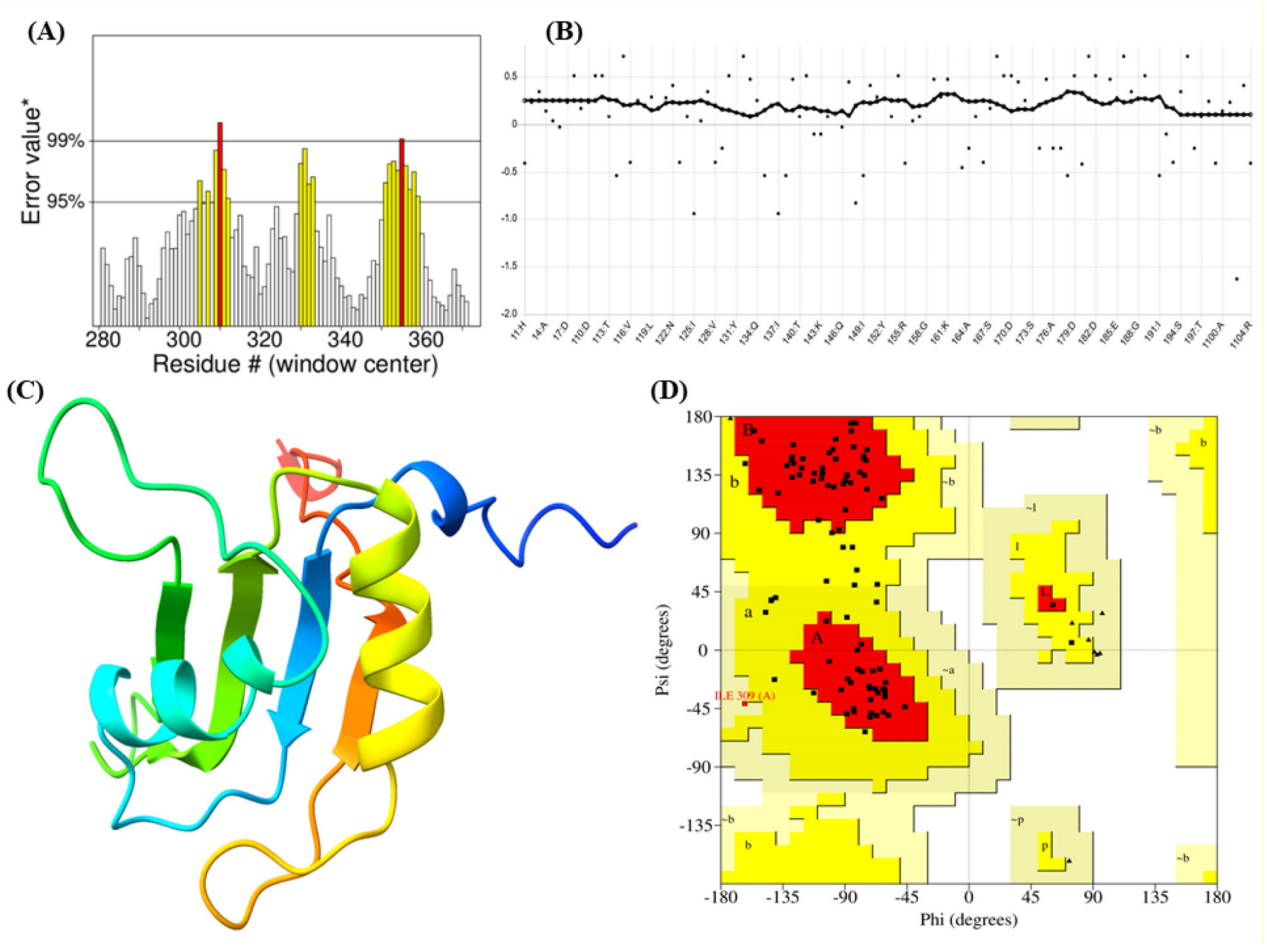
Integrated analysis of protein dynamics and stability: **(A, B)** Residue-wise metric indicating local properties along the protein sequence. **(C)** Three-dimensional ribbon representation of the protein structure highlighting secondary structural elements. **(D)** Ramachandran plot illustrating the distribution of backbone dihedral angles (phi and psi), with dense regions indicating preferred conformational states.

The Ramachandran plot of the I-TASSER model revealed that 80.0% of the residues were in the most favorable regions, with only 1 residue in a less favorable region and none in disallowed regions. This contrasts sharply with the initial AlphaFold2 and SwissModel predictions. Additionally, the ERRAT score improved to 79.12, indicating a much higher degree of structural integrity. The Verify3D assessment further confirmed that 98.08% of the residues had favorable 3D-1D compatibility, suggesting that the protein's 3D structure was well-aligned with its primary sequence (Figure 1, Table 1). Thus, while AlphaFold2 and SwissModel are powerful tools, they may struggle with proteins like FUS that exhibit highly flexible or loop-rich regions. I-TASSER's combination of threading, structural assembly, and refinement yielded a superior model in this case, making it the most suitable choice for downstream analyses.

**Table 1:** Comparative validation scores for FUS protein model.

**Validation metric**	**AlphaFold2**	**SwissModel**	**I-TASSER**
ERRAT score	59.8	65.3	79.12
Verify3D score (%)	82.5%	85.7%	98.08%
Ramachandran favored (%)	70.0%	74.0%	80.0%
ProSA Z-Score (recommended)	−2.5	−1.8	−3.0
QMEAN score (recommended)	0.68	0.72	0.80

### 3.2 ML-based virtual screening

#### 3.2.1 Dataset compilation and refinement

The main dataset used for this study consisted of 1,038 unique compounds (Supplementary Table S1). Out of these, 463 were found to be active against the FUS proteins. The remaining 575 compounds were used as decoy molecules. We thoroughly checked the dataset to ensure its accuracy, with no missing values or duplicate entries, making it suitable for further analysis. During the refinement stage, we examined the molecules in detail by converting their SMILES representation into computational descriptors using the RDKit software package. This allowed us to extract 33 relevant features, which will form the basis of our future research (Supplementary Table S1). The molecular descriptors used in this study were key to predicting how compounds interact with the FUS protein. LogP was included to assess the hydrophobicity of each compound, with values below 5 indicating a good balance for crossing the blood-brain barrier (BBB). Molecular Weight (MW), kept under 500 Daltons, was crucial for ensuring good bioavailability and cellular penetration. The number of Hydrogen Bond Donors and Acceptors was minimized to improve BBB penetration, as fewer hydrogen bonds generally enhance a compound's ability to enter the brain. Additionally, Topological Polar Surface Area (TPSA) was used, with lower values (below 90 Å^2^) favoring better bioavailability and BBB permeability. Together, these descriptors ensured that the selected compounds were not only effective in interacting with the FUS protein but also possessed favorable pharmacokinetic properties for drug development.

#### 3.2.2 Analysis of active inhibitors against FUS protein using machine learning

**Figure 2:**
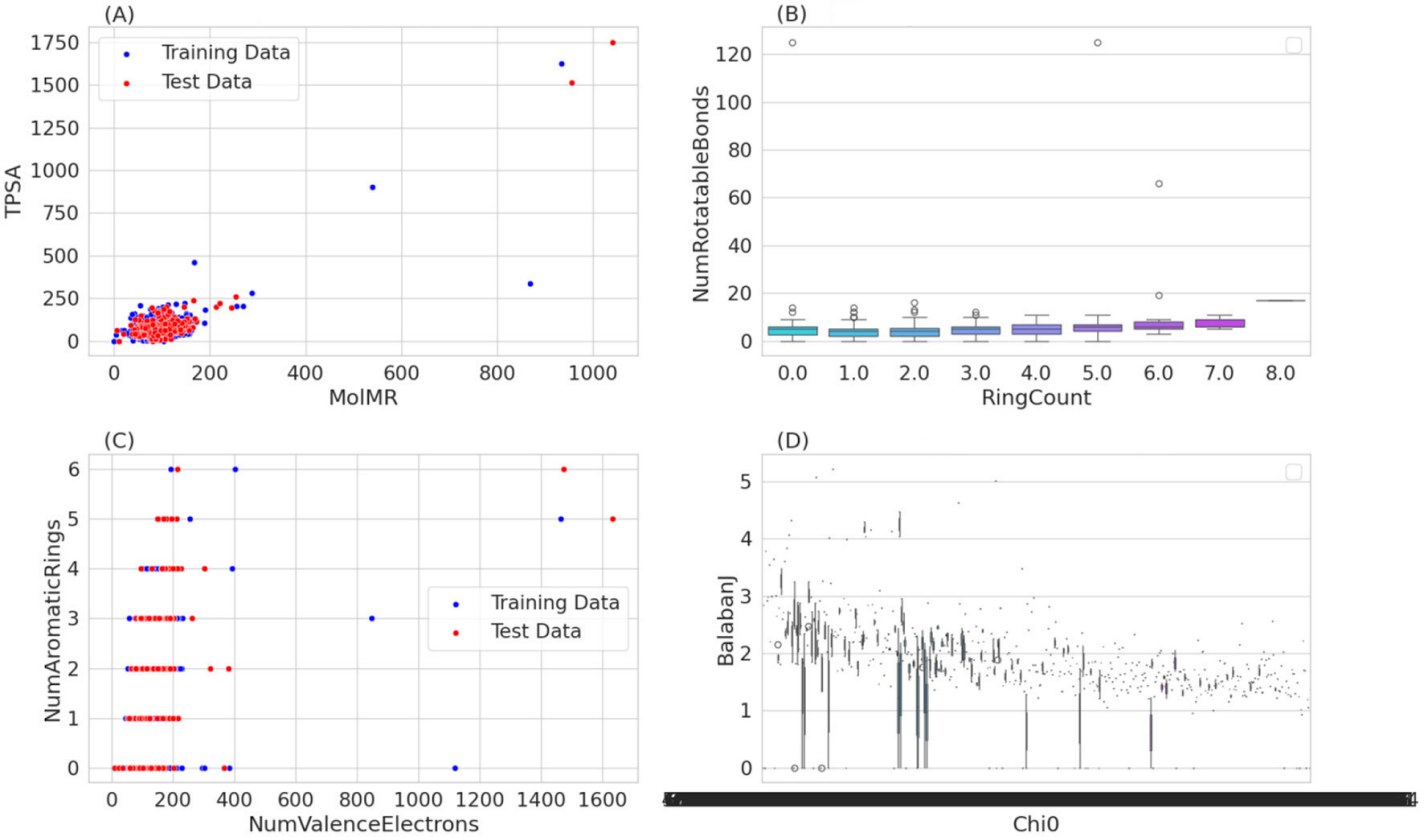
Provides a multi-faceted graphical analysis of chemical compounds, comparing training and test data based on various molecular descriptor. Scatter plots illustrate the relationship between molecular refractivity and polar surface area, and the correlation between valence electrons and aromatic ring count, revealing trends and outliers in the dataset. A box plot details the distribution of rotatable bonds across different ring counts, indicating variability that does not strongly correlate with the ring count. Lastly, a scatter plot comparing the Chi0 and Balaban J indices suggests a complex relationship between these molecular connectivity descriptors. **(A)** MoIMR vs. TPSA. **(B)** RingCount vs. NumRotatableBounds. **(C)** NumValenceElectrons vs. NumAromaticRings. **(D)** Chi0 vs. Balaban J.

The transformed data was split into a training set and a test set (Figure 2). In our effort to classify active inhibitors targeting FUS proteins, we used various machine learning methods: k-Nearest Neighbors (kNN), Support Vector Machines (SVM), Random Forests (RF), and Naive Bayes (NB). These models were built using the sklearn library in Python, with data obtained from the Binding DB repository. Their efficacy was critically assessed through a range of evaluative criteria, such as accuracy, recall (or sensitivity), specificity, Matthews Correlation Coefficient (MCC), and the Area Under the Curve (AUC).

**Figure 3:**
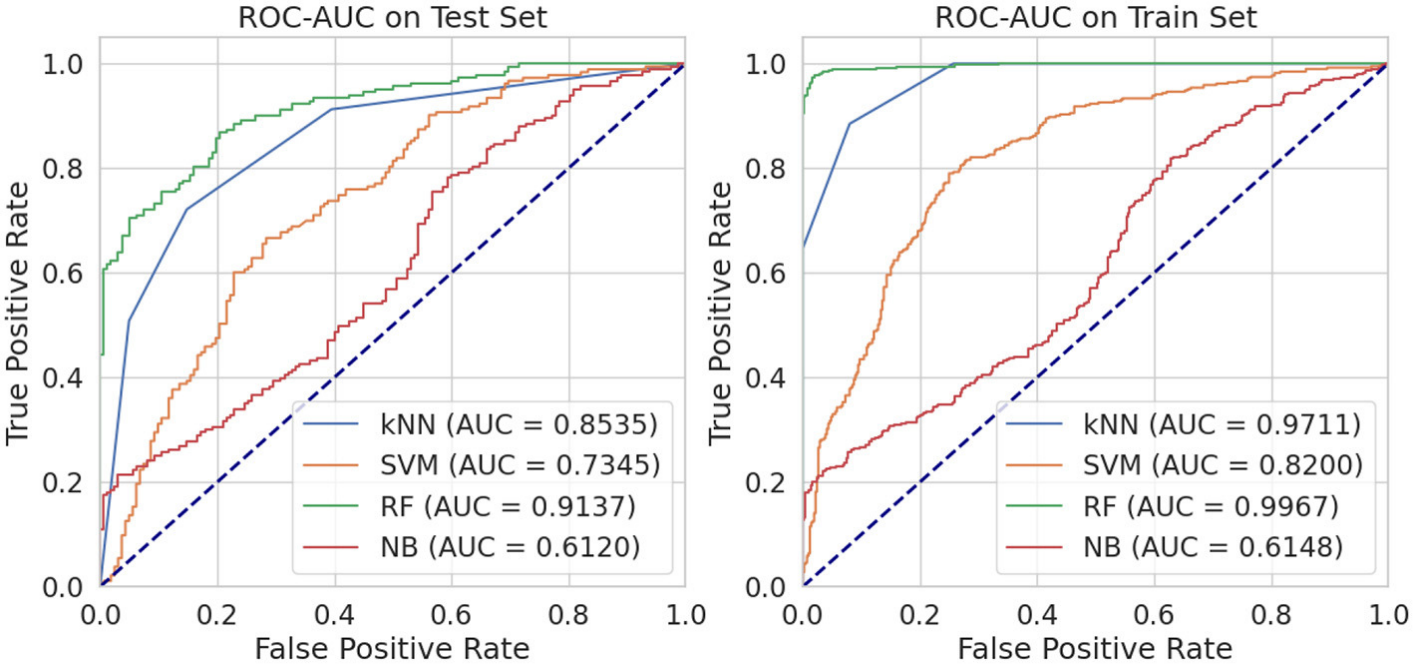
Performance comparison of four machine learning classifiers: k-Nearest Neighbors (kNN), Support Vector Machines (SVM), Random Forest (RF), and Naive Bayes (NB).

On the test set, we evaluated four different models using a metric called AUC (Area Under the Curve), which measures their ability to classify data accurately. The RF classifier achieved an impressive AUC of 0.9137, indicating a high true positive rate across different thresholds while maintaining a relatively low false positive rate (Figure 3). The kNN model performed well with an AUC of 0.8535, followed by the SVM with an AUC of 0.7345. However, the NB model had the lowest AUC of 0.6120, suggesting it was less effective in accurately classifying the given data. When we examined the training set, we found similar results with the RF model outperforming the others with an AUC of 0.9967, which is close to perfect. The kNN model also showed excellent performance with an AUC of 0.9711, while the SVM had a lower yet significant AUC of 0.8200. Again, the NB model had the lowest AUC of 0.6148.

Based on these findings, RF model consistently performed better than the other models in terms of AUC for both the training and testing sets. This superior performance can be attributed to RF's ensemble nature, which reduces overfitting by combining predictions from multiple decision trees. Additionally, RF handles imbalanced datasets effectively and identifies the most important features, making it well-suited for chemical datasets like ours. In contrast, SVM relies heavily on tuning parameters, which can limit its performance if not optimized. Naive Bayes assumes feature independence, which is not ideal for chemical descriptors, leading to lower accuracy. kNN was moderately successful but is sensitive to the choice of neighbors and distance metrics, which can affect stability in high-dimensional data. Thus, the Random Forest model's ability to accurately classify molecules based on their properties makes it an ideal choice for virtual screening of phytochemicals as natural inhibitors against FUS protein. By using this model, researchers can efficiently identify potential candidates from a large pool of compounds, saving time and resources in the initial stages of drug development.

### 3.3 Screening of phytocompounds library

**Figure 4:**
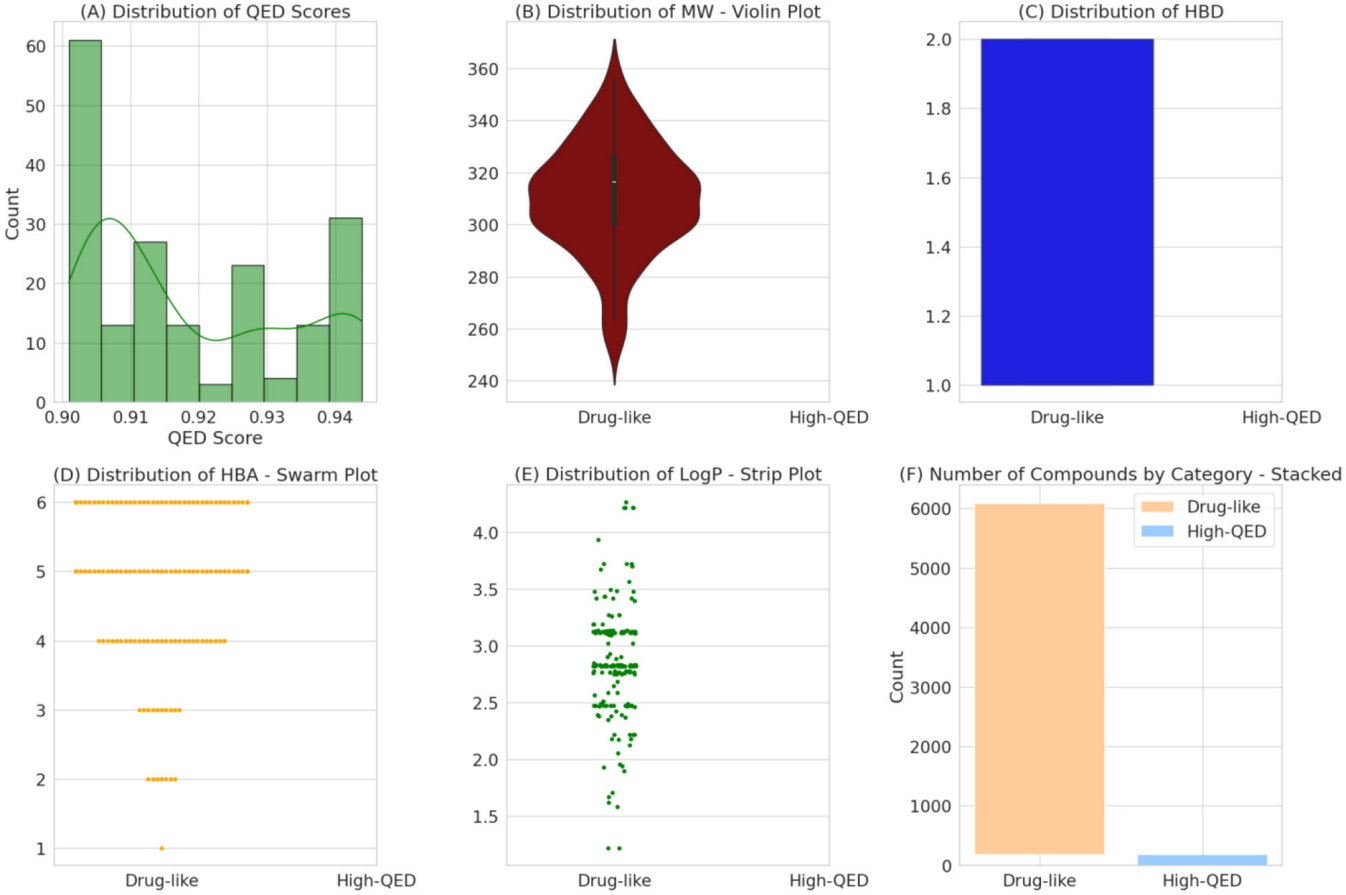
A multi-panel display characterizes the physicochemical properties of drug-like and high-QED compounds. Histograms and density plots **(A)** reveal the distribution of QED scores, while violin and bar plots **(B, C, F)** compare the molecular weights and counts of compounds in each category. Swarm and strip plots **(D, E)** provide detailed distributions for hydrogen bond acceptors and lipophilicity (LogP) respectively, delineating the spread of these properties within the dataset.

Using the power of the RF model, we carefully reviewed FDA-approved drugs for neurodegenerative diseases to see if they could be effective against the FUS protein. We screened a large number of drugs and selected 9,583 based on specific criteria (Supplementary File 3). The way a drug moves through the body, including how it's absorbed, distributed, metabolized, and excreted (ADME), is influenced by its own molecular properties. Certain factors like molecular weight (MW), number of hydrogen bond donors, number of hydrogen bond acceptors, and the octanol-water partition coefficient (LogP) play a crucial role in determining how a drug works and how effective it can be. From our analysis, we consistently found that the chosen drug candidates had Molecular weights under 500 Daltons. This indicates an optimal size for easy entry into cells and better availability in the body (Figure 4). The selected compounds had <5 hydrogen bond donors: These drugs have fewer components that can form hydrogen bonds with other molecules. They also have fewer sites where they can accept hydrogen bonds from other molecules. Lastly, the LogP values under five shows a good balance between being soluble in both water and fat, which helps with distribution in the body. All these characteristics make these drugs more likely to interact effectively with biological molecules and be absorbed well by the body. They also have an ideal balance between hydrophilicity and lipophilicity, which is important for their distribution in different parts of the body.

The RF model then assessed these drugs, and intriguingly, 5,903 of them were predicted to have a potential active interaction with the FUS protein, as detailed in Supplementary File 4. Further after applying Threshold on QED value, a few were selected for docking analysis. The prediction that nearly 6% of the screened drugs might be effective against FUS is significant.

### 3.4 Molecular docking analysis

**Figure 5:**
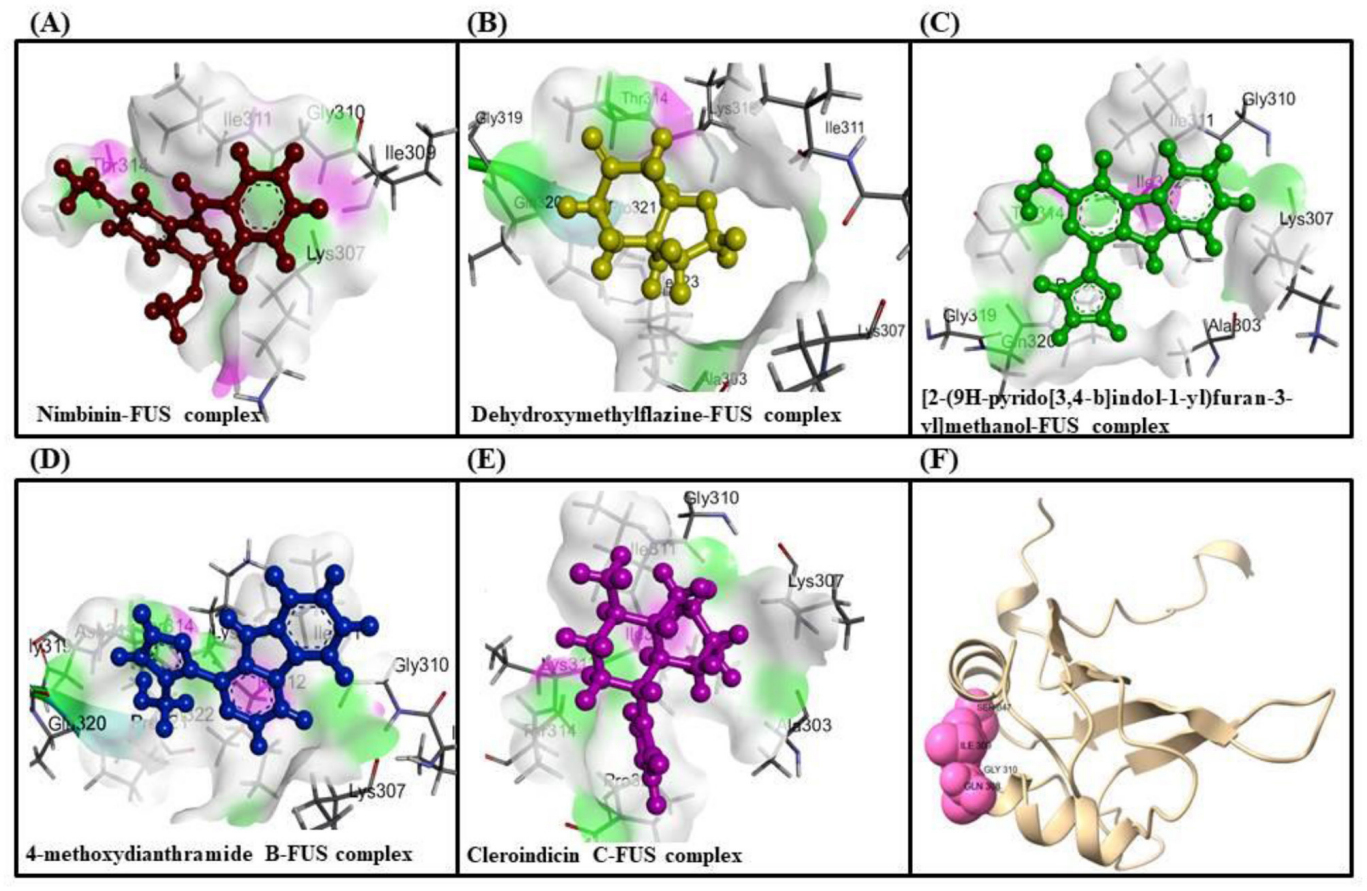
Showcases molecular docking poses of various compounds within the binding sites of the FUS protein, as predicted by the CASTp tool. **(A–E)** Depict the molecular interactions between FUS and the compounds nimbinin, dehydroxymethylflazine, [2-(9H-pyrido[3,4-b]indol-1-yl)furan-3-yl]methanol, 4-methoxydianthramide B, and cleroindicin C, respectively, with each compound rendered in a distinct color and the amino acid residues involved in the interaction labeled. **(F)** Highlights the binding pockets on the FUS protein surface as determined by CASTp, emphasizing the potential sites of interaction for the displayed compounds.

Molecular docking analysis were conducted by using software AutoDock Vina through the PyRx virtual screening tool to determine the interaction between the different compounds with the FUS protein. The CASTp tool was used to determine the binding pocket, as shown in Figure 5 and Table 2. The docking results revealed the binding efficiencies and stabilities of different compounds with the FUS protein. Compound nimbinin showed a high binding affinity i.e., 9.74 kcal/mol with RMSD 1.42 Å implies a consistent docking pose. Dehydroxymethylflazine was even found to have a higher binding affinity i.e., 10.17 kcal/mol with RMSD 1.59 Å suggested a high interaction with slightly higher structural deviation upon binding. The complex molecule [2-(9H-pyrido[3,4-b]indol-1-yl)furan-3-yl]methanol presented a binding affinity of 9.13 kcal/mol and an RMSD of 1.85 Å, which may reflect a secure yet less defined binding conformation. 4-methoxydianthramide B demonstrated a binding affinity of 8.95 kcal/mol and exhibited the lowest RMSD at 1.02 Å among the tested compounds, indicating a highly congruent fit within the FUS protein's active site. Cleroindicin C identified with a substantial binding affinity of 10.12 kcal/mol and the smallest RMSD value of 0.77 Å, implying an exceptionally precise interaction with the protein's binding pocket. In summary, the docking studies suggest that Dehydroxymethylflazine and Cleroindicin C have significant potential as FUS protein inhibitors, given their strong binding affinities and low RMSD values, which are indicative of stable and precise binding.

**Table 2:** Binding affinity and RMSD values of top five phytocompounds against FUS protein.

**Compound names**	**Binding affinity (kcal/mol)**	**RMSD (Å)**	**2D structure**
Nimbinin	9.74	1.42	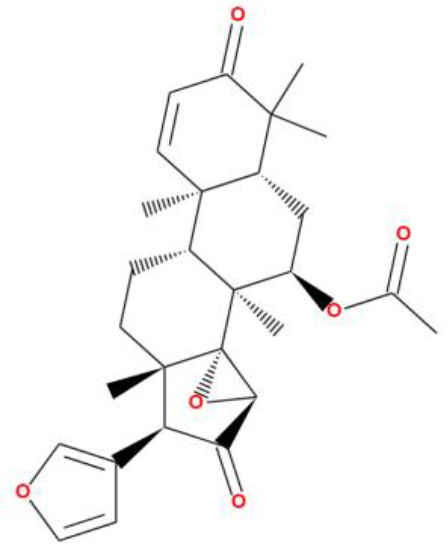
Dehydroxymethylflazine	10.17	1.59	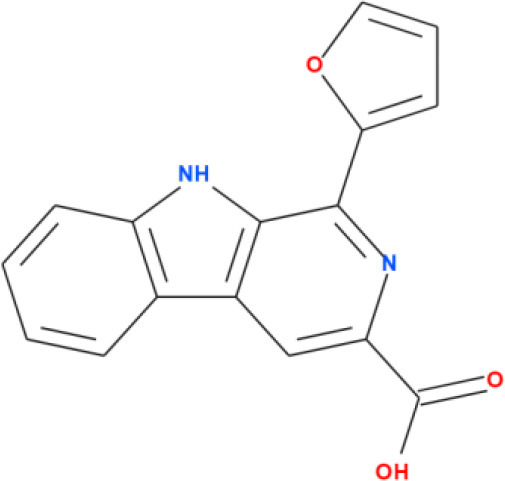
[2-(9H-pyrido[3,4-b]indol-1-yl)furan-3-yl]methanol	9.13	1.85	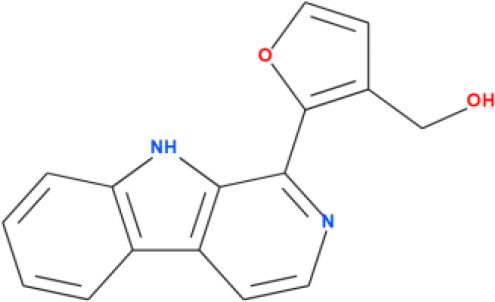
4-methoxydianthramide B	8.95	1.02	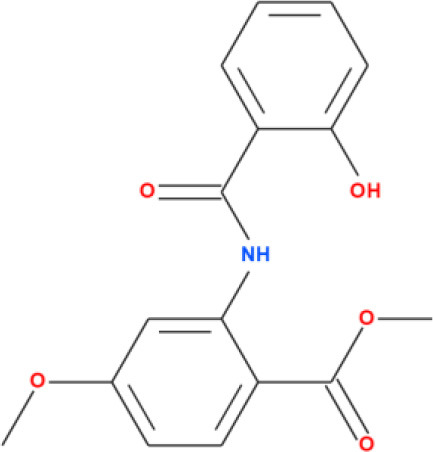
Cleroindicin C	10.12	0.77	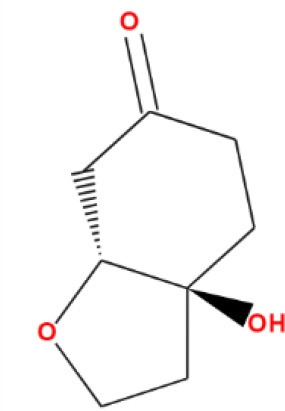

### 3.5 MD simulation analysis

**Figure 6:**
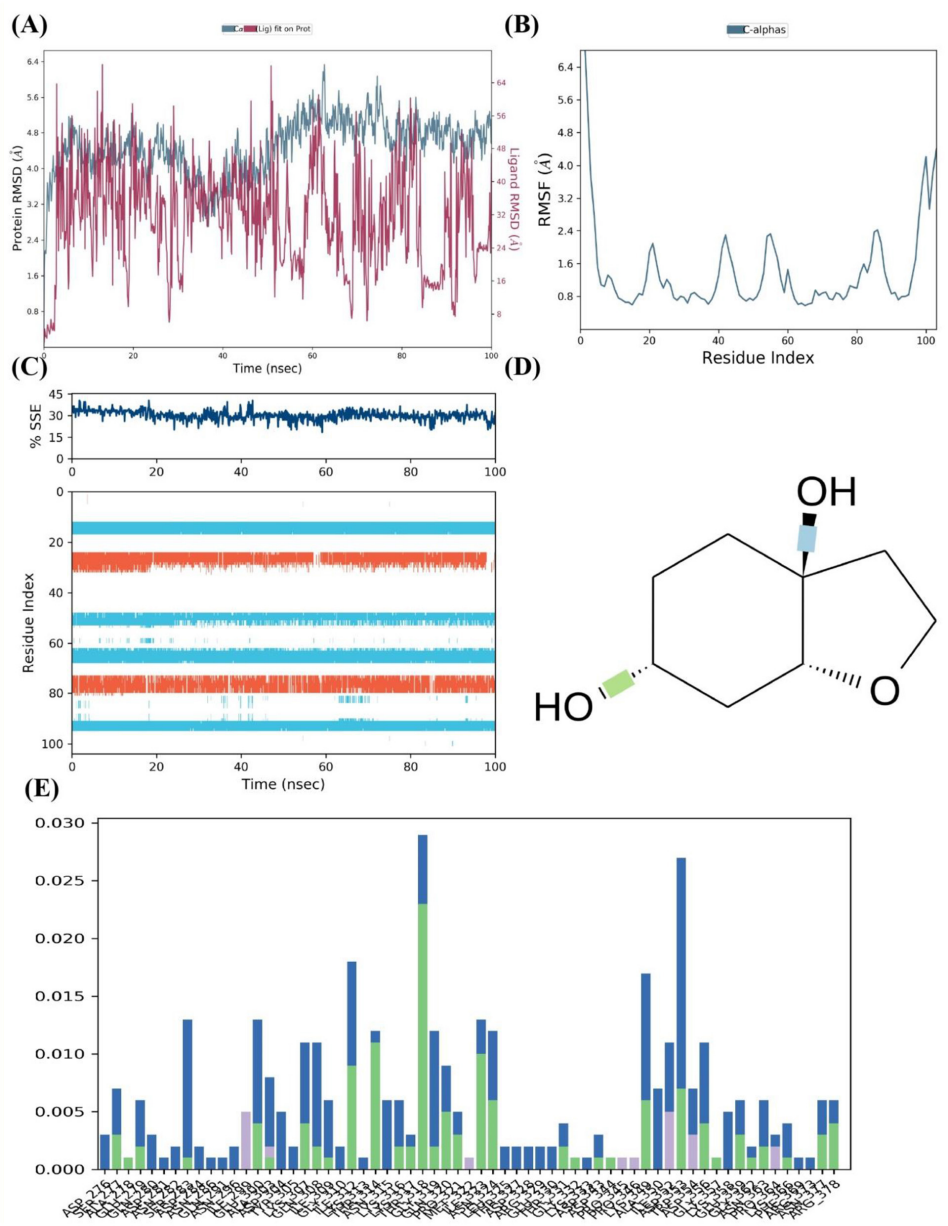
MD Simulation Analysis of FUS Protein Interaction with Cleroindicin C. **(A)** After 100 ns of simulation, the RMSD of the C-alpha atoms of the FUS protein with (blue) and without (red) cleroindicin C. **(B)** Flexible areas are highlighted by the FUS protein residues' RMSF. **(C)** The percentage of structural stability with time for β-sheets (orange) and α-helices (blue) is shown by the SSE. **(D)** Chemical structure of cleroindicin C. **(E)** The frequency of interactions between cleroindicin C and FUS protein residues; the height of the bars indicates the proportion of simulation time along ligand' contact.

The FUS protein's MD simulations in the presence of cleroindicin C provide many insights into the complex's stability and binding affinity. A comparison of the structural deviations of the protein with and without the ligand bound is demonstrated by the analysis of the protein RMSD (Root Mean Square Deviation) across the 100 ns simulation period, as shown in Figure 6A. Following an initial equilibration period, the ligand-bound FUS protein's RMSD values appear to oscillate between around 2.0 and 4.5 Å, indicating a stable complex. On the other hand, the unbound protein exhibits larger variations, which may indicate decreased stability when cleroindicin C is not present.

The RMSF for the C-alpha atoms across the residue index are shown in Figure 6B. The graph's peaks indicate areas of the protein structure that are more flexible. Multiple peaks above 4.0 Å RMSF may indicate flexible loops or domains that are important for the protein's interaction with cleroindicin C or for the protein's function. Figure 6C shows a steady percentage of SSE during the simulated time frame, with largely unchanged α-helix and β-sheet content. The stability observed in secondary structural elements may indicate that the protein retains its original shape when it binds to the ligand. The frequency of interactions between the FUS protein residues and cleroindicin C is displayed in the residue-wise interaction plot shown in Figure 6E. The bars show the percentage of the simulation time that every residue spends with the ligand within a certain interaction distance. Remarkably, some residues show a greater interaction frequency, suggesting important contact sites that probably support cleroindicin C's strong affinity for binding the FUS protein.

Lastly, the molecular structure of cleroindicin C, as shown in Figure 6D, implies that the compound has functional groups that can form hydrophobic contacts, hydrogen bonds, or pi-pi stacking, which could further support the binding affinity found in the MD simulation results. Together, these results suggest a stable interaction between FUS and cleroindicin C, with potential implications for the protein's function and the therapeutic utility of the compound. The MD simulation demonstrates not only the stability of the complex but also identifies critical residues that could be essential for the high affinity binding of cleroindicin C to the FUS protein.

**Figure 7:**
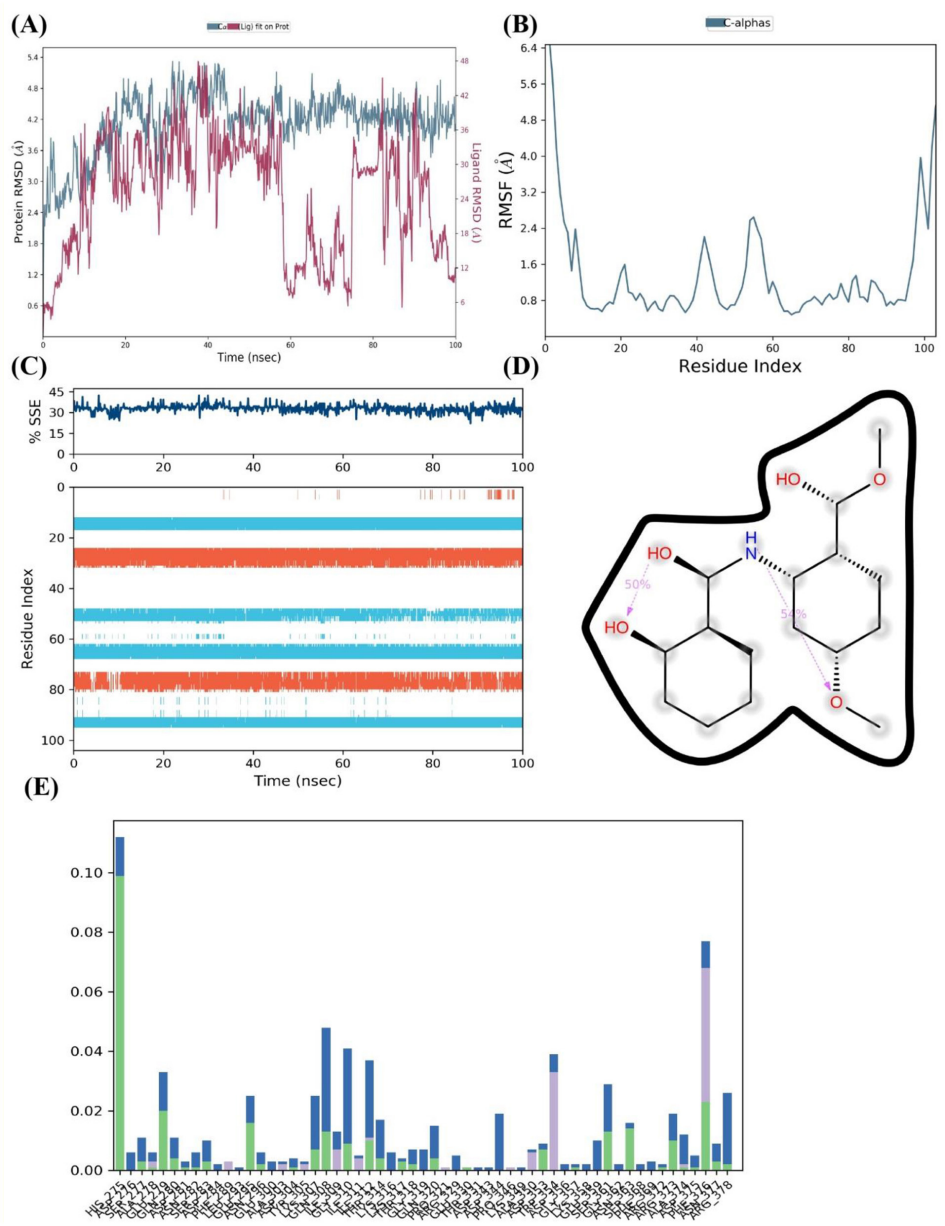
4-methoxydianthramide B binding insights into the FUS protein by MD simulation. **(A)** The FUS protein's RMSD across a 100 ns simulation, with (blue) and without (red) the ligand 4-methoxydianthramide B, demonstrating the stability of the protein-ligand interaction. **(B)** RMSF across residues of the FUS protein, demonstrating a constant stiffness with particular flexible areas. **(C)** The percentage of SSE in the FUS protein during the simulation, showing how the structure is preserved when a ligand is bound. **(D)** The chemical structure of 4-methoxydianthramide B with dotted lines indicating potential hydrogen bonding interactions. **(E)** A residue interaction frequency map showing the interaction between the 4-methoxydianthramide B and the FUS protein.

After an initial period of equilibration, the RMSD values for the FUS protein in the presence of 4-methoxydianthramide B show a trend of stabilizing, according to MD simulation. As can be observed in Figure 7A, the protein-ligand complex's RMSD trajectories fluctuate within a range that implies a stable association throughout the simulation. Compared to the broader oscillations seen in the absence of the ligand, the ligand-bound protein RMSD values fluctuate around a more compact band, indicating that the ligand's presence may add extra stability to the protein structure.

The RMSF for the C-alpha atoms in the FUS protein is shown in Figure 7B. The smaller frequency of variations across most residue indices indicates a protein structure that is solid and sustained upon ligand binding. This could indicate that 4-methoxydianthramide B binds closely within the binding site. A few peaks can be seen, indicating certain flexible areas that may be crucial for the dynamic character of the protein-ligand interaction. Over the course of the simulation, the FUS protein's SSE, as seen in Figure 7C, remained rather constant. The preservation of α-helices and β-sheets indicates that the protein's overall secondary structure remains intact upon ligand interaction.

Figure 7D shows the highlighted chemical structure of 4-methoxydianthramide B. Annotations (shown as dotted lines) suggest possible hydrogen bond interactions that may be important for the binding process. Potential hydrogen bonds imply that the protein and ligand bind at certain high-affinity locations. Finally, Figure 7E shows the frequency of contact between 4-methoxydianthramide B and the residues of the FUS protein. The bars in this Figure 7 indicate the percentage of the simulation period that the residues are in close proximity to the ligand. A notable frequency of interaction is displayed by certain residues, suggesting that these residues may serve as important points of contact within the ligand-binding domain. Therefore, a scenario where 4-methoxydianthramide B shows a good binding affinity with the FUS protein is supported by the MD simulation data. This conclusion is supported by the protein-ligand complex's stability, the retention of protein secondary structure, the specificity of ligand-residue interactions, and the possibility of strong hydrogen bonding.

**Figure 8:**
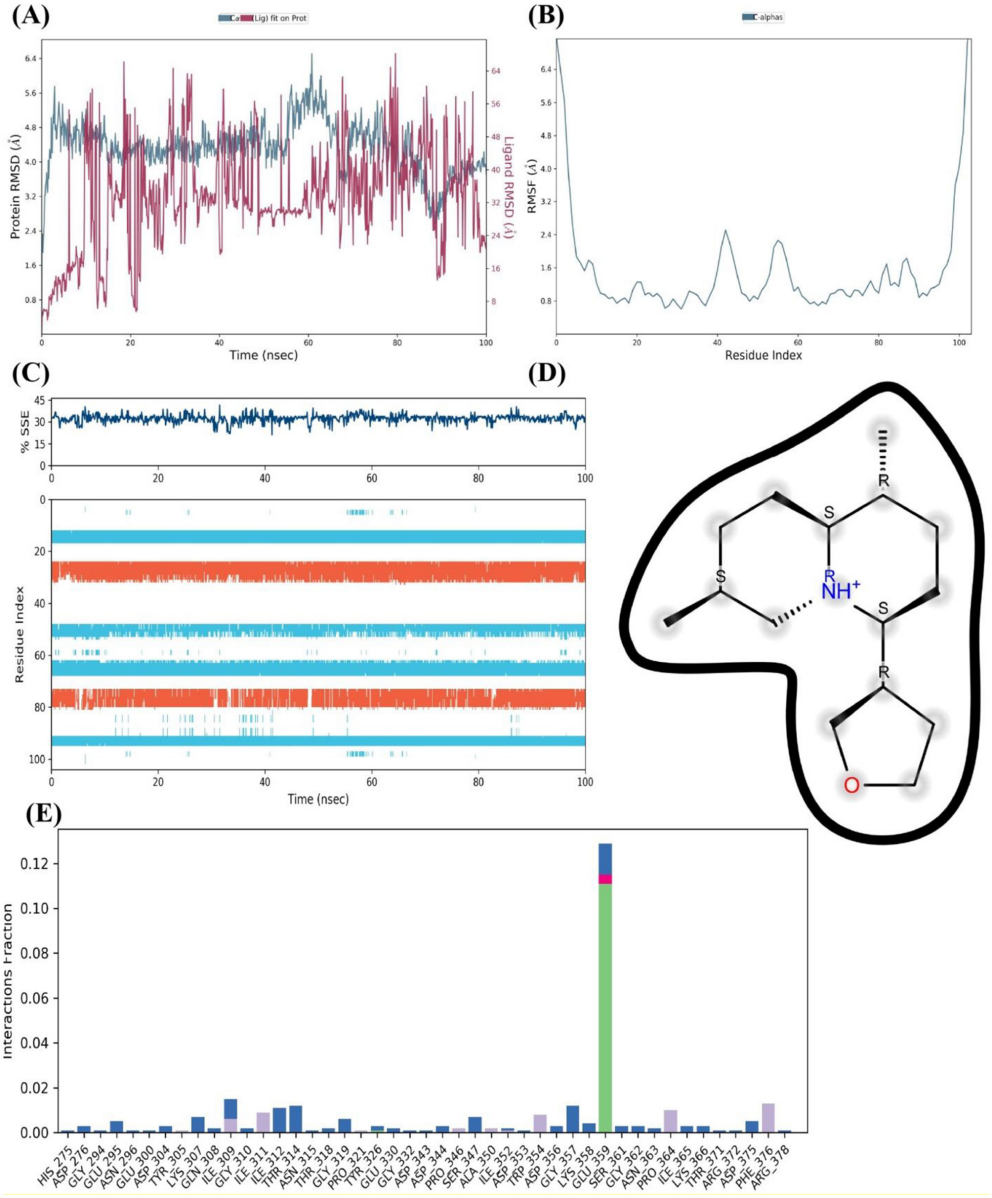
The FUS Protein and Nimbinin MD simulation' results. **(A)** RMSD trajectories of the FUS protein over 100 ns showing a stable protein-ligand combination with (blue) and without (red) nimbinin. **(B)** Regions of flexibility are highlighted by the RMSF for C-alpha atoms across the protein residues. **(C)** The simulation timeline's consistency in the proportion of secondary structure elements (SSEs) demonstrates the structural integrity of the FUS in the presence of nimbinin. **(D)** Nimbinin's chemical structure, showing possible hydrogen bonding positions with annotations. The interaction frequency plot **(E)** illustrates the fraction of interaction between particular residues of the FUS protein and nimbinin.

Further, insights into the interaction between the FUS protein and nimbinin was gained through simulation of the protein complex on a 100 nanosecond timescale. The RMSD for the FUS protein alone and in association with nimbinin is shown in Figure 8A. Following an initial phase of adjustment, the protein-ligand complex, represented by the blue line, exhibits RMSD values indicating a stable connection. When compared to the protein without nimbinin, the overall RMSD values stay below 5.5 Å and show fewer deviation spikes, indicating that the ligand helps to maintain the protein's structure. The RMSF for the protein residues' C-alpha atoms in Figure 8B demonstrates a variation that varies across the protein chain. Less flexibility and greater rigidity are indicated by lower RMSF values, which tend to occur when nimbinin is present. There are multiple peaks in the Figure 8, which represent areas where the protein's structure is more flexible by nature or where nimbinin binding may cause conformational changes.

As shown Figure 8C, the SSE % does not change much throughout the course of the simulation, suggesting that nimbinin does not substantially alter the FUS protein's overall secondary structure. This shows that the substance may stabilize the current structure rather than denature the protein. The molecular structure of nimbinin is depicted in Figure 8D, highlighting possible hydrogen bonding positions that are necessary for potent and targeted interactions with the protein. Finally, the interaction proportion of nimbinin with individual FUS protein residues is shown in Figure 8E. The graph shows which residues interact with nimbinin most frequently; these residues may represent important binding sites. Remarkably, a few residues exhibit an exceptionally high interaction proportion, suggesting their potential significance in nimbinin's binding affinity to the FUS protein. As a result, the MD simulation results suggest that the FUS protein and nimbinin have a persistent and possibly robust binding relationship. Sustained RMSD values, steady SSE percentages, and particular residue interactions with nimbinin are among the evidence that lend credence to the theory that nimbinin has a good binding affinity for the FUS protein, which may have consequences for the compound's potential therapy.

**Figure 9:**
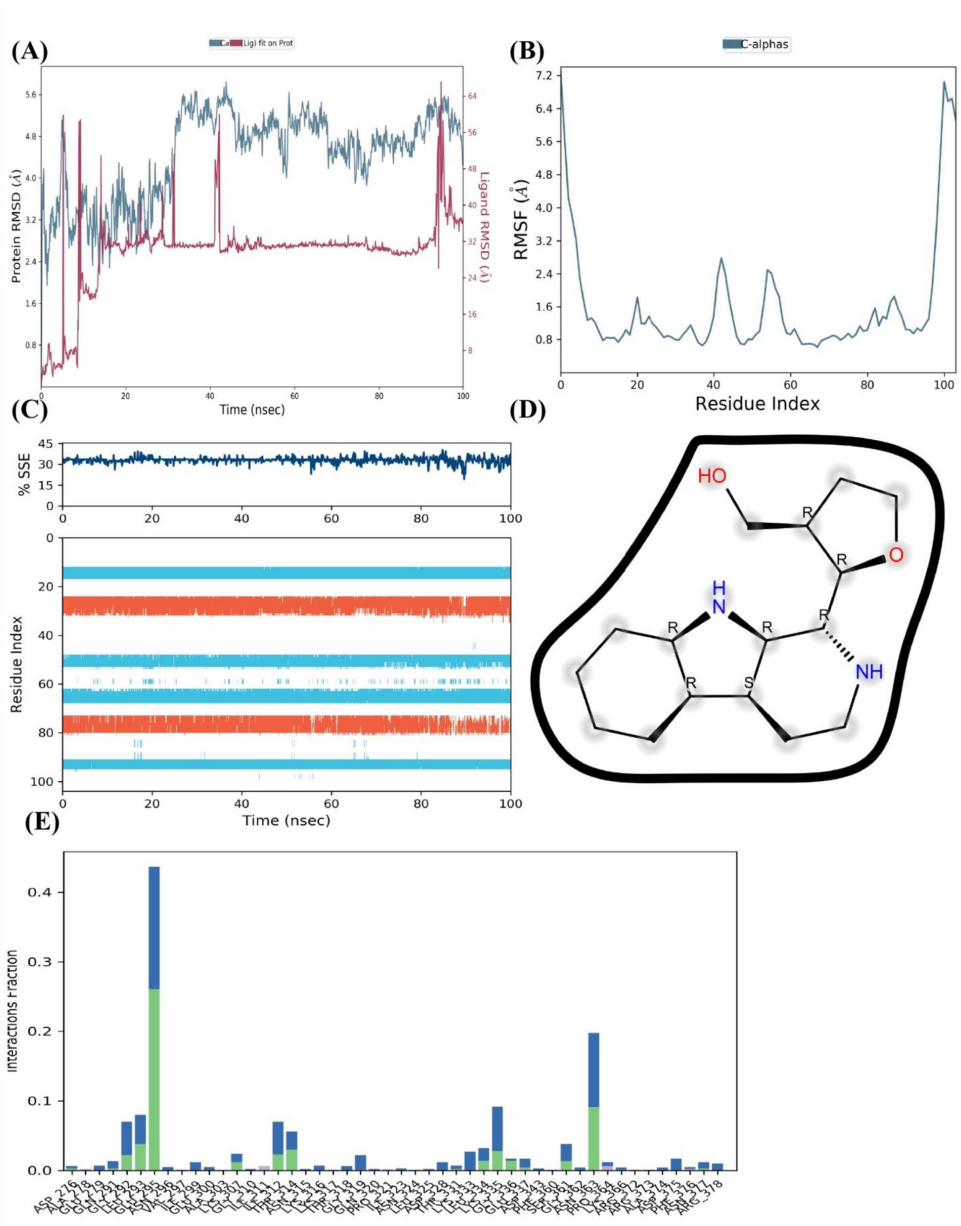
MD Simulation of the Interaction Between FUS Protein and [2-(9H-pyrido[3,4-b]indol-1-yl)furan-3-yl]methanol. **(A)** RMSD analysis of FUS protein with the ligand (blue) and without (red) over 100 ns, indicating a stable complex. **(B)** RMSF profile for C-alpha atoms of FUS protein residues, identifying flexible regions. **(C)** Percentage of secondary structure elements of FUS protein, showing the preservation of structure upon ligand binding. **(D)** Chemical structure of [2-(9H-pyrido[3,4-b]indol-1-yl)furan-3-yl]methanol with potential hydrogen bonding interactions marked. **(E)** Interaction fraction of FUS protein residues with the ligand, with significant interactions highlighted, suggesting key residues involved in binding.

Throughout the course of 100 nanoseconds, a complete understanding of the FUS protein in complex with [2-(9H-pyrido[3,4-b]indol-1-yl)furan-3-yl]methanol is provided by the MD simulation. The RMSD of the protein is shown in Figure 9A both with and without the ligand (blue and red). Following an initial rise, the ligand-bound protein's RMSD shows a stabilization tendency, with values primarily staying below 3 Å, indicating a stable protein-ligand combination following equilibration. On the other hand, the protein lacking the ligand exhibits larger swings in RMSD values, which would indicate reduced stability in the ligand-free state.

The RMSF across the residues of the protein shows diversity with a few conspicuous peaks in Figure 9B. These peaks show areas of the protein that are more dynamic or flexible, which may be important for protein function or ligand binding.

The SSE %, as shown in Figure 9C, does not change throughout the course of the simulation, indicating that the protein's overall secondary structure is preserved when the ligand is present. This structural stability is important because it indicates that the native shape of the protein is not considerably perturbed by ligand interaction. The chemical structure of the ligand is depicted in Figure 9D, emphasizing possible sites of interaction like donors and acceptors of hydrogen bonds. These positions most likely play a crucial role in important interactions that support the stability of the binding with FUS protein residues.

Last but not least, Figure 9E's interaction fraction plot illustrates how frequently distinct protein residues bind with the ligand. It is possible that some residues are essential for the ligand's binding affinity to the FUS protein since they show a greater interaction fraction, which denotes a stronger or more consistent interaction with the ligand. Together, the simulated results point to a successful binding of [2-(9H-pyrido[3,4-b]indol-1-yl)furan-3-yl]methanol to the FUS protein, as demonstrated by the stable RMSD, the maintained secondary structure, and the interactions between certain residues. These results suggest that the compound has a promising binding affinity for the FUS protein, which may indicate its potential therapeutic relevance.

**Figure 10:**
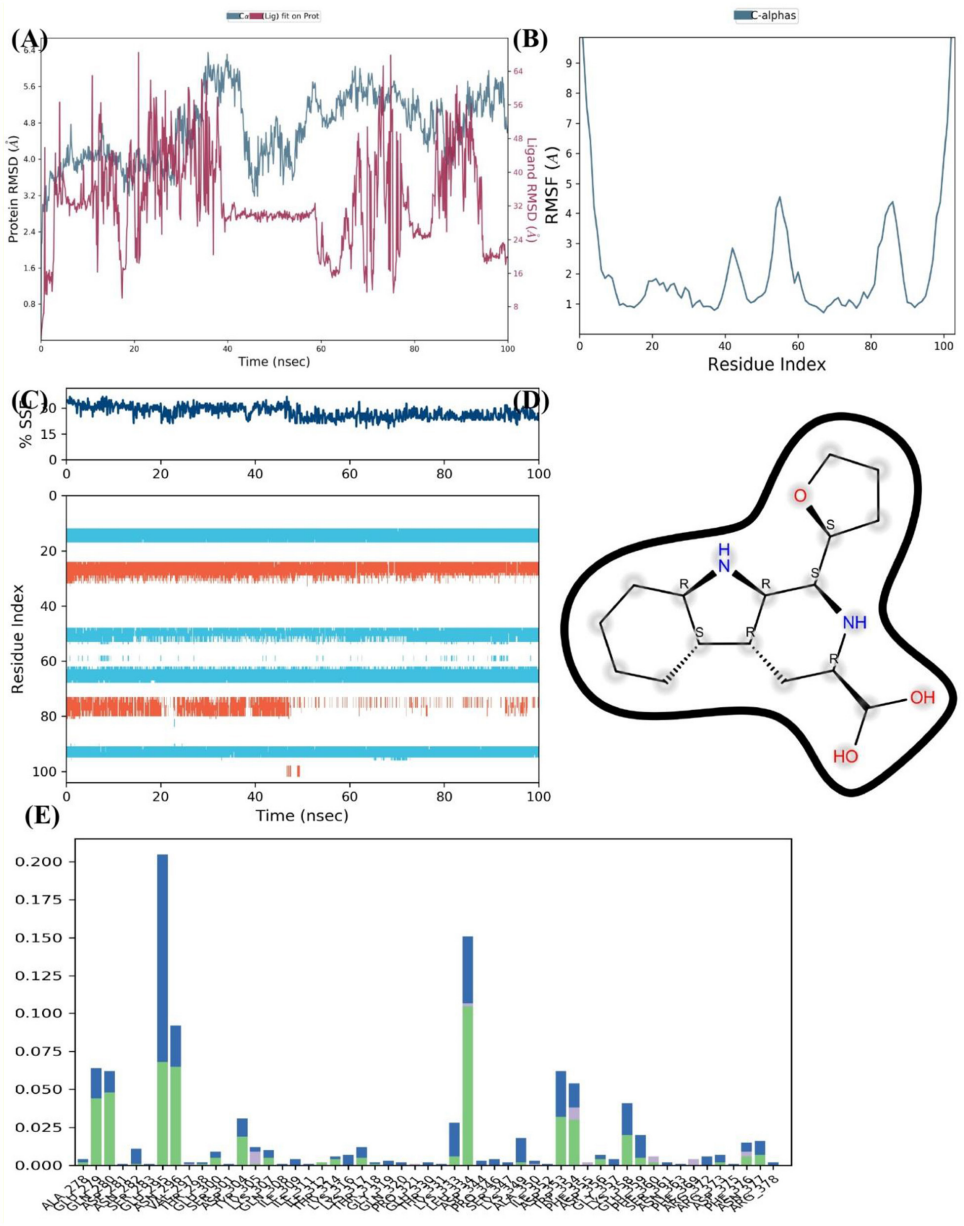
MD Simulation of FUS Protein Interaction with Dehydroxymethylflazine. **(A)** Protein RMSD values over 100 ns for the FUS protein with (blue) and without (red) Dehydroxymethylflazine, indicating the ligand's stabilizing effect. **(B)** RMSF of FUS protein C-alpha atoms highlighting regions of flexibility and potential ligand interaction sites. **(C)** Consistent percentages of SSEs across the simulation, suggesting structural integrity of the protein in complex with the ligand. **(D)** Chemical structure of Dehydroxymethylflazine with potential interaction points for hydrogen bonding. **(E)** Residue interaction fraction plot for the FUS protein, identifying residues with higher frequencies of interaction with Dehydroxymethylflazine, suggesting critical binding regions.

The MD Simulation results for the FUS protein complexed with Dehydroxymethylflazine over a 100 nanosecond period provide substantial evidence of interaction and stability. From Figure 10A, the RMSD analysis for the protein both with and without the ligand indicates a period of stabilization post-initial equilibration. The protein-ligand complex RMSD levels off after about 20 ns, suggesting that Dehydroxymethylflazine may contribute to a stable conformation of FUS. Notably, the complex shows a similar RMSD range to the unbound protein, which might imply that the ligand does not cause significant conformational stress.

The RMSF depicted in Figure 10B reveal specific regions of the FUS protein that exhibit increased mobility. These fluctuations, particularly those with higher RMSF values, might indicate regions of the protein that are involved in the binding or are affected by the presence of the ligand. Further, the percentage of SSE throughout the simulation, displayed in Figure 10C, remains consistent for the protein, showing no significant loss of structural elements like alpha helices or beta sheets. This constancy suggests that the protein's secondary structure is not adversely affected by binding with Dehydroxymethylflazine.

In Figure 10D, the chemical structure of Dehydroxymethylflazine is presented, highlighting the functional groups that could be involved in key interactions with the FUS protein, such as hydrogen bonds, indicated by the dashed lines. Figure 10E shows the interaction fraction of FUS protein residues with the ligand. Certain residues show higher interaction frequencies, denoting that these are likely to be the critical contact points within the binding pocket of the protein where Dehydroxymethylflazine may form stable interactions. Overall, the MD simulation results suggest that Dehydroxymethylflazine exhibits a potential for good binding affinity with the FUS protein. This is based on the ligand's ability to maintain a stable RMSD, the absence of disruptive effects on the protein's secondary structure, and specific interactions with key protein residues. These factors are promising for considering Dehydroxymethylflazine as a compound that can effectively interact with the FUS protein.

## 4 Discussion

Dementia encompasses a family of neurodegenerative diseases with multifactorial and largely poorly understood origin with substantial social impact. FUS (Fused in Sarcoma) is a nuclear protein with a strong RNA binding capacity and a crucial regulator of many processes of RNA metabolism within neural cells. Mutations in the gene and its transport to the cytoplasm lead to formation of pathological aggregates of FUS which are linked to neuronal toxicity *in vivo*. These insights indicate that FUS holds a potential to become a therapeutic target for dementia related disorders. In this study, we analyzed of the partial structure of the FUS protein using AlphaFold2 and SwissModel. The model revealed a primarily loop heavy tertiary protein structure. Loops represent regions of the protein which potentially sample a large number of possible conformations and therefore cannot always be well determined spatially by structural modeling. Our analysis of the ERRAT and Verify3D scores for the initial protein structure raised concerns of the reliability of the predicted structure. Subsequently more advanced models of protein structure were generated using the more reliable I-TASSER suite yielding a model with much better Rao-Z score, ERRAT, and Verify3D scores. This powerful model was then used as a basis for the following docking studies. To further improve our approach, we turned to machine learning methods to screen through a large set of potential inhibitors. Using the Random Forest (RF) algorithm, which proved itself a better performer than other tested methods in predicting the efficacy of the compounds, we finally selected nimbinin, dehydroxymethylflazine, [2-(9H-pyrido[3,4-b]indol-1-yl)furan-3-yl]methanol, 4-methoxydianthramide B, and cleroindicin C. RF achieved excellent AUCs for both training (0.9967) and testing (0.9137) sets and played an important role in integrally selecting the compounds for further study.

The molecular docking results obtained using AutoDock Vina with the assistance of CASTp binding pocket predictions indicated that compounds could form strong specific interactions with the FUS protein. In particular, dehydroxymethylflazine and cleroindicin C showed higher binding affinities, 10.17 and 10.12 kcal/mol, respectively, as well as RMSD values, indicating a stable binding conformation. Based on the molecular docking results, these two compounds were regarded as foremost candidates and possible natural inhibitors of the FUS protein. In previous research, the findings were in line with the potential of the aforementioned compounds. The chemical profiles of these potential compounds were in line with drug-likeness and bioavailability and would be useful in a physiological environment. The compounds exhibited a molecular weight below 500 Daltons, fewer than 5 hydrogen bond donors, and LogP values, which were less than five, thereby ensuring favored pharmacokinetic profiles.

Dehydroxymethylflazine and Cleroindicin C are phytochemicals with a more recent focus toward potential therapeutic interventions against multiple diseases and disorders. Sourcing from two unique plants of medicinal importance, the phytochemical compound Dehydroxymethylflazine is derived from the plant's of the genus Scutellaria, notably the Scutellaria baicalensis. Scutellaria is a known source of flavonoids and specifically baicalin, which has previously been investigated for its neuroprotection. However, a more focused analysis on the utility of Dehydroxymethylflazine has seemingly been scarce. In the context of neurodegenerative disease, demonstrated Dehydroxymethylflazine activity is thus also an adjunct.

Cleroindicin C comes from the Clerodendrum indicum plant, which is used in Ayurvedic medicine and has a wide range of effects. While research has looked at extracts from Clerodendrum helping protect neurons, there hasn't been much specific work on Cleroindicin C for neurodegenerative diseases. This makes it an interesting area to study more. Both Dehydroxymethylflazine and Cleroindicin C exhibit promising drug-like properties that align with the “rule of five,” a guideline used to predict a compound's absorption and permeability in the human body. Their molecular weights, each below 500 daltons, suggest that they could effectively penetrate cells and achieve bioavailability. Additionally, the limited number of hydrogen bond donors and acceptors further supports their capacity for favorable solubility and membrane permeability, which are critical factors for oral drug formulations. Moreover, their lipid solubility values fall within the optimal range, increasing the likelihood of crossing the blood-brain barrier, an essential characteristic for drugs targeting neurological conditions. Recent studies have demonstrated that both compounds exhibit strong affinities for the FUS protein, indicating their potential to interact with and possibly inhibit FUS dysfunction, which is linked to dementia. The RMSD values from these studies suggest that these interactions may be stable. Given their favorable pharmacokinetic profiles and preliminary evidence of interaction with the FUS protein, Dehydroxymethylflazine and Cleroindicin C appear to be strong candidates for further research. With the growing demand for novel treatments for neurodegenerative diseases, exploring therapeutic compounds derived from traditional plants through modern drug discovery techniques holds considerable promise in this complex and challenging field.

Finally, through long-duration MD simulations lasting 50 ns, we have confirmed the stability of the interaction between the compounds and the FUS protein. The consistent affinity observed throughout these simulations supports the docking findings and strengthens the potential of these compounds as therapeutic candidates. Our study's robustness is grounded in the collaboration of machine learning models, thorough data curation, molecular docking, and dynamic simulations. By pinpointing compounds such as dehydroxymethylflazine and cleroindicin C that exhibit strong binding affinities and stable interactions with the FUS protein, we are pushing the boundaries of drug discovery in dementia. This brings us closer to a future where the regulation of FUS protein activity could potentially slow down the progression of the disease. The combination of computer-based and experimental techniques represents a cutting-edge approach to drug discovery. Our research demonstrates the effectiveness of using computational methods to speed up the initial phases of drug development, especially when dealing with challenging targets such as FUS linked to dementia. The predictive models and simulations utilized in this study provide a more efficient way to identify potential inhibitors. With additional testing and improvements, these findings could lead to innovative treatments for dementia.

## 5 Conclusion

In this study, we explored a computational approach to discover natural inhibitors for the FUS protein, a key target in neurodegenerative diseases such as dementia. Our refined structural model of the FUS protein, achieved through I-TASSER, overcame the limitations of initial predictions from AlphaFold2 and SwissModel, providing an accurate framework for further analysis. Through machine learning, specifically the Random Forest algorithm, we identified promising phytochemicals such as nimbinin, dehydroxymethylflazine, and cleroindicin C. Molecular docking results, performed using AutoDock Vina, highlighted dehydroxymethylflazine and cleroindicin C as compounds with strong binding affinities and stable interactions with the FUS protein. Molecular dynamics simulations further validated these results by confirming the stability of these interactions. Additionally, the identified compounds exhibited favorable pharmacokinetic profiles, suggesting their potential to penetrate the blood-brain barrier, a critical factor for neurodegenerative treatment development. The broader implications of this research lie in the identification of plant-derived compounds with the potential to inhibit the FUS protein, opening new pathways for natural product-based drug development for dementia. This computational approach provides a foundation for future experimental validation and clinical exploration, encouraging a shift toward sustainable, natural therapies in neurodegenerative research. In future work, experimental studies should focus on validating the identified compounds' efficacy and safety. Furthermore, advanced molecular dynamics simulations could enhance the understanding of protein-ligand interactions, paving the way for optimization of these natural inhibitors.

## Data Availability

The raw data supporting the conclusions of this article will be made available by the authors upon request.
